# HPV-driven metabolic reprogramming promotes T cell exhaustion: targeting immunometabolic barriers with natural products

**DOI:** 10.3389/fimmu.2026.1892919

**Published:** 2026-07-23

**Authors:** Liang Wei, Hou Menghui, Zhang Dandan, Wang Xin, Chu Xinying, Ma Guangyi, Ma Jing

**Affiliations:** 1 First Teaching Hospital of Tianjin University of Traditional Chinese Medicine, Tianjin, China; 2 National Clinical Research Center for Chinese Medicine, Tianjin, China

**Keywords:** cervical cancer, human papillomavirus, metabolic reprogramming, natural products, T cell exhaustion, tumor microenvironment

## Abstract

**Background:**

High-risk human papillomavirus (HR-HPV) infection is the primary driver of cervical cancer. Emerging evidence indicates that the tumor microenvironment (TME) undergoes severe metabolic rewiring, which may accelerate T cell exhaustion (TEX) and impair immune checkpoint blockade (ICB). However, the molecular mechanisms coupling HPV-driven metabolism to T cell dysfunction remain incompletely elucidated.

**Methods:**

This review summarizes immunometabolic interactions in the cervical cancer TME. We examined the metabolic alterations induced by HPV oncoproteins (E6/E7) and how they reshape nutrient availability, lactate accumulation, and lipid peroxidation to drive anti-tumor CD8^+^ T cell exhaustion.

**Results:**

HPV-driven aerobic glycolysis and IDO1-mediated tryptophan catabolism establish severe metabolic barriers, causing nutrient deprivation and histone lactylation in infiltrating lymphocytes. These alterations are associated with persistent mitochondrial stress and ferroptosis, accelerating terminal TEX. In preclinical models, natural products (e.g., curcumin, berberine, quercetin, and artemisinin derivatives) can counteract this immunosuppressive rewiring by targeting checkpoints such as HIF-1α, PKM2, and AMPK; however, direct evidence of CD8^+^ tumor-infiltrating lymphocyte rescue in HPV-specific immune-competent systems remains limited, largely inferred from other tumor types. Nanomedicine delivery may further enhance the bioavailability and targeting of these herbal components.

**Conclusion:**

HPV-induced metabolic reprogramming is proposed to act as a fundamental checkpoint driving T cell exhaustion in cervical cancer. Targeting these immunometabolic barriers using natural compounds, particularly via nanomedicine-based delivery strategies, represents a promising but still largely preclinical avenue to synergize with conventional immunotherapies and potentially overcome resistance.

## 1 Introduction

Persistent infection with high-risk human papillomavirus (HR-HPV) is the principal etiological driver of cervical carcinoma and a spectrum of anogenital malignancies, collectively accounting for over 600,000 new cancer cases annually worldwide (1). Although prophylactic vaccines have substantially reduced incident HR-HPV infection in vaccinated cohorts, therapeutic options for established invasive disease remain limited, particularly in the recurrent or metastatic setting. Immune checkpoint blockade (ICB), exemplified by programmed cell death protein 1/programmed death-ligand 1 (PD-1/PD-L1) inhibitors, has achieved regulatory approval for advanced cervical cancer. Nevertheless, objective response rates in this indication remain modest, ranging from approximately 15% to 25% in unselected populations (2), and durable remissions are confined to a minority of patients. This pronounced clinical heterogeneity implies that immunosuppressive mechanisms operating beyond canonical receptor–ligand checkpoint interactions sustain immune evasion in HPV-associated malignancies, rendering ICB monotherapy insufficient to restore productive antitumor immunity in most cases.

A growing body of evidence identifies T cell exhaustion (TEX) as the central pathological correlate of ICB failure, and metabolic reprogramming of the tumor microenvironment (TME) as a principal driver of this dysfunctional state (3, 4). HPV oncoproteins E6 and E7 co-opt the host metabolic machinery to enforce the Warburg effect—aerobic glycolysis despite adequate oxygen tension—satisfying the bioenergetic demands of viral replication and accelerated cell proliferation (5). The ensuing metabolic remodeling creates a TME characterized by severe glucose deprivation, lactate accumulation, tryptophan catabolism via indoleamine 2,3-dioxygenase 1 (IDO1), and progressive hypoxia. These perturbations collectively impose profound metabolic stress on tumor-infiltrating lymphocytes (TILs): disruption of mitochondrial dynamics, epigenetic silencing of effector gene programs through histone lactylation, and heightened susceptibility to ferroptosis driven by lipid peroxidation ultimately render TILs metabolically unfit and terminally exhausted (6, 7). Critically, these metabolic barriers are thought to persist even when PD-1 signaling is pharmacologically blocked, providing a mechanistic rationale for the suboptimal efficacy of ICB monotherapy and positioning metabolic checkpoint targeting as an attractive complementary strategy to overcome ICB resistance. Because this integrated model draws on evidence of varying strength, throughout the review we distinguish mechanisms directly demonstrated in HPV-positive cervical cancer from those extrapolated from other tumor types, chronic-infection models, or general immunometabolism, using hedged language where a mechanism has not been directly established in HPV-associated cervical cancer tumor-infiltrating lymphocytes (TILs); the corresponding evidence level is indicated in the summary tables and figure legends.

Natural products have attracted considerable attention as candidate metabolic checkpoint modulators in this context. Unlike conventional cytotoxic agents that inflict indiscriminate cellular damage, bioactive phytochemicals offer a complementary metabolic pharmacology: certain compounds preferentially target oncometabolic enzymes—such as hexokinase 2 (HK2), pyruvate kinase M2 (PKM2), lactate dehydrogenase A (LDHA), and indoleamine 2,3-dioxygenase 1 (IDO1)—to reduce nutrient competition within the TME, thereby indirectly relieving the metabolic pressure on TILs; others directly rehabilitate exhausted TILs by restoring mitochondrial biogenesis through the SIRT1/PGC-1α axis or by suppressing lipid peroxidation-driven ferroptosis (8). A subset of agents—notably berberine and curcumin—show preliminary evidence of acting on both tumor and immune compartments, though simultaneous dual-compartment validation in co-culture or immune-competent *in vivo* models remains lacking for most agents. Whether a given compound acts primarily on the tumor compartment, the immune compartment, or both is therefore agent-specific and context-dependent, a nuance that critically informs the therapeutic window analyses presented in this review.

Despite this promise, prior reviews have addressed HPV oncobiology, T cell exhaustion, and natural product pharmacology largely in isolation. This review integrates recent evidence linking HPV-associated tumor metabolism, T cell exhaustion, and natural product-based metabolic intervention, and systematically evaluates phytochemicals as agent-specific metabolic modulators—acting on the tumor compartment, the immune compartment, or both—in this specific tumor context. We systematically examine HPV-driven metabolic barriers in the TME, the resulting intrinsic TIL metabolic dysfunctions, natural product-based interventions organized by metabolic target axis, and translational challenges with proposed biomarker-stratified trial designs.

## 2 Immunosuppressive metabolic microenvironment in HPV-associated malignancies

The persistence of HR-HPV infection is not sustained solely by viral antigen variation but is thought to be further enabled, at least in part, by virus-directed metabolic remodeling of the local tissue microenvironment. HPV-transformed cells avidly compete for available nutrients while continuously exporting immunosuppressive metabolic byproducts—most prominently lactate—into the pericellular space. The resulting milieu, characterized by substrate depletion and accumulation of metabolic toxins, is proposed to constitute overlapping physical and biochemical barriers that promote the metabolic exhaustion of infiltrating CD8^+^ T cells before effective antigen clearance can occur; it should be noted that much of the supporting evidence is extrapolated from broader cancer immunometabolism rather than derived directly from HPV-positive cervical cancer TILs (9). The following subsections systematically delineate four mechanistically distinct layers of this metabolic immunosuppression: Warburg-driven glucose competition, lactate accumulation and microenvironmental acidification, lactate-mediated epigenetic consolidation of T cell exhaustion, and IDO1-dependent tryptophan catabolism. Hypoxia-amplified immunosuppression, which intersects and reinforces each of these axes, is addressed below.

### 2.1 Warburg effect-mediated nutrient immunosuppression

Under the influence of HPV oncoproteins E6 and E7, cervical epithelial cells undergo a fundamental metabolic reprogramming: even in oxygen-sufficient conditions, they preferentially rely on aerobic glycolysis for energy production—a phenomenon known as the Warburg effect. Crucially, this E6/E7-orchestrated metabolic adaptation serves not only the bioenergetic demands of rapid viral replication but also evolves into an active strategy of immune evasion, establishing the oncoproteins as the central upstream drivers of the immunosuppressive metabolic microenvironment.

At the molecular level, HPV E6/E7 oncoproteins upregulate glucose transporter 1 (GLUT1) and HK2 through multiple converging signaling axes, dramatically increasing cellular glucose uptake capacity (10). Beyond the canonical hypoxia-inducible factor-1α (HIF-1α)-dependent pathway, Hu et al. (5) demonstrated that HPV E6/E7 stabilizes oncogenic MYC mRNA through upregulation of the m^6^A reader protein IGF2BP2, markedly amplifying intracellular MYC protein levels and sustaining a high-flux glycolytic phenotype. This malignant metabolic reprogramming endows HPV-transformed tissue with an overwhelming competitive advantage in glucose uptake, rapidly depleting extracellular glucose and rendering infiltrating immune cells metabolically incapable of mounting productive effector responses (11, 12).

For effector T cells, glucose serves not merely as a bioenergetic substrate but as an essential signaling molecule. Early T cell receptor (TCR) activation critically depends on glycolysis-derived phosphoenolpyruvate (PEP) to sustain endoplasmic reticulum Ca²^+^-ATPase activity, thereby driving NFAT transcription factor nuclear translocation (13, 14). In the HPV-induced hypoglycemic microenvironment, impaired PEP synthesis attenuates Ca²^+^ influx and NFAT signaling, preventing transcriptional activation of key antiviral cytokines including IFN-γ and TNF-α (15). Compounding this, substrate-deprived GAPDH translocates to bind IFN-γ mRNA, suppressing its translation (16). Glucose deprivation thus coordinately suppresses T cell effector function at both transcriptional and translational levels, establishing it as the primary metabolic checkpoint of antitumor immunity in HPV-associated TME.

### 2.2 Lactate accumulation and microenvironmental acidification

The accelerated glycolytic activity driven by HPV infection generates a massive flux of lactate, which is continuously exported into the extracellular space via monocarboxylate transporter 4 (MCT4), causing a marked reduction in local microenvironmental pH (17). This acidification is far more than a physicochemical change; it directly subverts T cell metabolic homeostasis through interference with lactate transport dynamics. Under physiological conditions, effector T cells rely on MCT1 to export their own glycolysis-derived lactate, maintaining intracellular pH stability. However, when extracellular lactate concentrations substantially exceed intracellular levels, the concentration gradient reverses, driving lactate influx into T cells. The resulting intracellular acidification inhibits phosphofructokinase activity, blocking glycolytic flux and thereby compromising both energy supply and effector function (18).

Beyond its direct metabolic effects, extracellular lactate exerts immunosuppressive actions by promoting the selective expansion of regulatory T cells (Tregs) within the TME. Unlike effector T cells, Tregs preferentially utilize lactate as an alternative carbon source through a Foxp3-dependent metabolic reprogramming that renders them comparatively insensitive to high-lactate, low-glucose conditions. This metabolic asymmetry further reinforces local immunosuppression and represents a critical barrier to ICB efficacy that checkpoint blockade alone cannot overcome.

### 2.3 Histone lactylation and epigenetic consolidation of T cell exhaustion

The accumulation of lactate in the TME exerts immunosuppressive effects that extend well beyond its metabolic consequences. Lactate functions as a direct substrate for a novel class of histone post-translational modification—lysine lactylation—that stably encodes the immunosuppressive metabolic state at the epigenetic level, establishing what may be termed metabolic-epigenetic memory of exhaustion (19). Lactate can enter the nucleus via MCTs and, under the enzymatic activity of acetyltransferases including p300 and the ACSS2–KAT2A complex, donate lactyl groups to histone lysine residues, thereby remodeling the chromatin landscape of infiltrating immune cells.

Single-cell sequencing data reveal significantly elevated histone H3 lysine 18 lactylation (H3K18la) levels in CD8^+^ TILs compared with their peripheral blood counterparts (20). This modification has been reported to be enriched at the promoter and enhancer regions of canonical exhaustion-associated genes including PDCD1, HAVCR2, TOX, and NFATC2, where it is associated with their transcription; importantly, H3K18la and H3K9la are best understood as context-dependent transcriptional regulators of CD8^+^ T cell function rather than as uniformly exhaustion-silencing marks (21). Whether H3K18la directly recruits the BAF (SWI/SNF) chromatin remodeling complex in exhausted CD8^+^ T cells has not, to our knowledge, been demonstrated, and we therefore do not posit a direct lactylation–BAF link. What is established is that chromatin-remodeling factors limit T cell persistence and shape the exhaustion program (22). These observations suggest that lactate-driven histone lactylation may contribute to the relative resistance of metabolically stressed TILs to surface-level ICB. We stress, however, that the role of lactylation is context-dependent: depending on antigen exposure, local lactate concentration, chromatin context, and T cell differentiation state, histone lactylation can be associated with effector, stem-like, or exhausted programs rather than acting as a one-directional “lock.” Consistent with this, physiological lactate concentrations have been reported to maintain CD8^+^ T cell stemness through histone lactylation, which cannot be reconciled with a purely suppressive, exhaustion-silencing model. Lactylation should therefore be regarded as a metabolite-driven, bidirectional transcriptional regulator whose net effect on TIL fate in the HPV-associated TME remains to be defined directly.

### 2.4 Tryptophan catabolism and IDO1-mediated immunosuppression

Dysregulated tryptophan metabolism represents another core mechanism by which HPV-associated tumors evade immune surveillance. The rate-limiting enzyme indoleamine 2,3-dioxygenase 1 (IDO1), which catabolizes the essential amino acid L-tryptophan along the kynurenine pathway, is aberrantly overexpressed in cervical cancer tissue and in multiple cell types within the HPV-associated TME. Inflammatory signals including IFN-γ—paradoxically released by activated T cells themselves—trigger negative-feedback IDO1 induction in tumor epithelial cells and antigen-presenting cells, converting an immune activation signal into a local immunosuppressive circuit (23).

The rapid depletion of tryptophan by IDO1 activates the nutrient-sensing kinase GCN2 within T cells, triggering the integrated stress response and broadly suppressing global protein translation, thereby inducing T cell proliferative arrest (24). Concurrently, the accumulating tryptophan catabolite kynurenine specifically activates the aryl hydrocarbon receptor (AHR) transcription factor, producing a second wave of immunosuppression: AHR signaling directly upregulates PD-1 surface expression on CD8^+^ T cells to accelerate their exhaustion (25), and simultaneously drives CD4^+^ T cell differentiation toward immunosuppressive FoxP3^+^ regulatory T cells (26). The IDO1–kynurenine–AHR axis thus builds a metabolic immunosuppressive barrier that synergizes with the glucose-competitive and lactate-acidification mechanisms described above, constructing a multi-layered checkpoint that comprehensively paralyzes antitumor immune responses and sustains persistent HPV infection.

### 2.5 Hypoxia and oxidative stress

As HPV-driven epithelial hyperproliferation outpaces angiogenesis during the progression from cervical pre-malignancy to invasive carcinoma, a local hypoxic microenvironment develops that both links metabolic reprogramming to immune evasion and acts as a key amplifier of local immunosuppression. Hypoxia stabilizes HIF-1α protein by inhibiting its ubiquitin-proteasomal degradation; nuclear HIF-1α then directly binds hypoxia response elements in the CD274 gene promoter, upregulating PD-L1 surface expression on HPV-infected cells and thereby strengthening inhibitory signaling toward effector T cells (27). Simultaneously, hypoxia-induced mitochondrial respiratory chain dysfunction generates excessive reactive oxygen species (ROS) accumulation within the TME (28). This oxidative stress disrupts T cell mitochondrial membrane potential, limiting ATP synthesis and impairing metabolic substrate utilization. Sustained oxidative damage also triggers DNA damage response pathways that compel a compensatory downregulation of the TCRζ chain—progressively eroding antigen recognition capacity and driving T cells toward terminal exhaustion (29).

**Figure 1:**
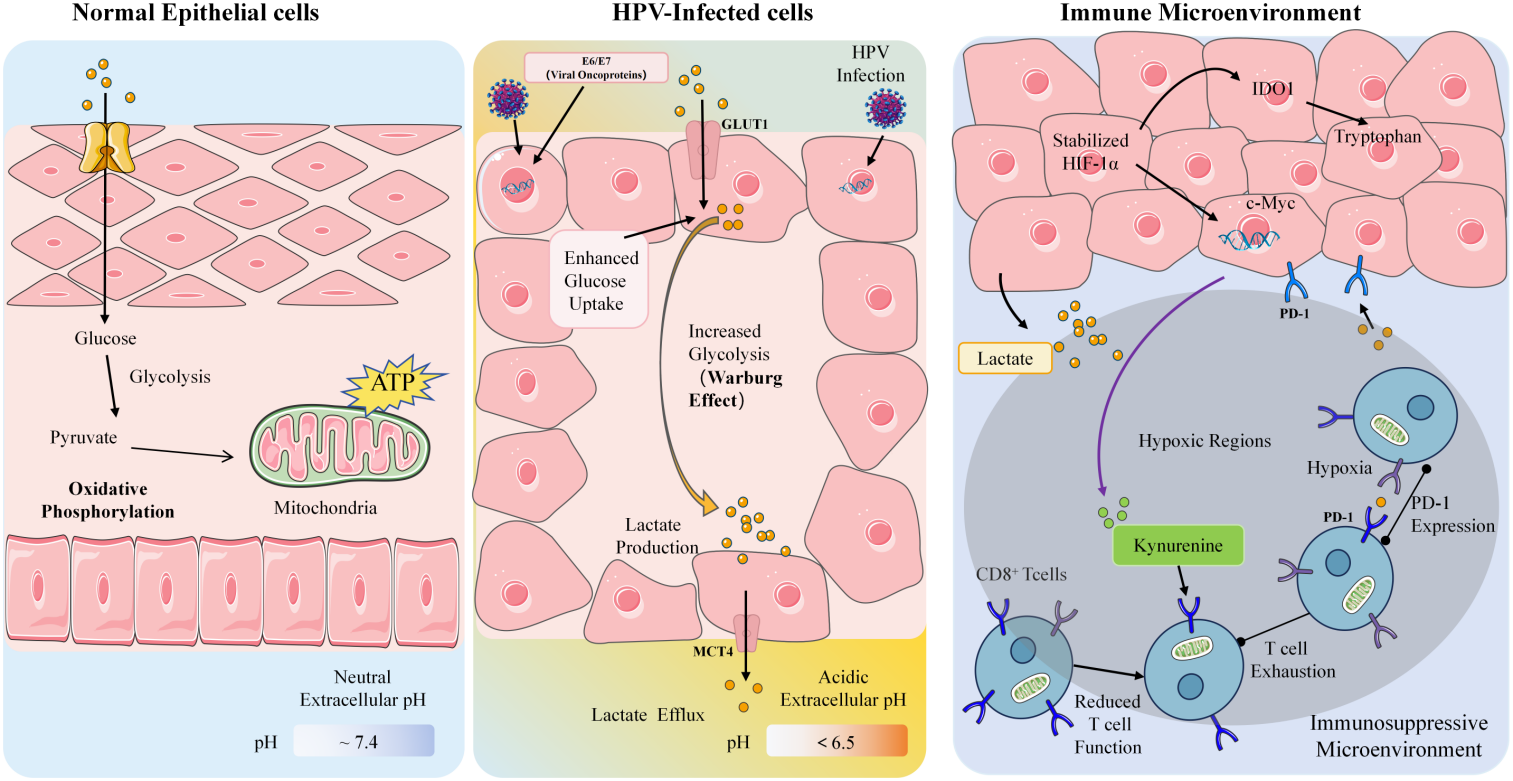
HPV E6/E7-mediated metabolic reprogramming and the formation of an immunosuppressive tumor microenvironment. HPV oncoproteins E6/E7 upregulate GLUT1 and HK2 via the IGF2BP2/MYC and HIF-1α axes, enforcing the Warburg effect. Accelerated glycolysis depletes extracellular glucose, exports lactate through MCT4 to acidify the microenvironment, and drives histone H3K18 lactylation that consolidates T cell exhaustion. IDO1 catabolizes tryptophan to immunosuppressive kynurenine, and hypoxia-stabilized HIF-1α upregulates PD-L1. Under this convergent pressure, infiltrating CD8^+^ TILs sustain mitochondrial dysfunction and terminal exhaustion. Evidence levels differ: the E6/E7–GLUT1/HK2 and IDO1/hypoxia–PD-L1 steps are supported by HPV/cervical studies, whereas the lactylation step and the depicted CD8^+^ TIL outcomes are largely extrapolated from non-HPV models and should be regarded as proposed (lactylation is context-dependent).

Collectively, HPV-encoded oncoproteins construct a metabolically hostile TME through coordinated suppression across four interconnected axes: glucose competition that starves TILs of the glycolytic substrate required for effector function; lactate-driven acidification and epigenetic consolidation that lock TILs into terminal exhaustion; IDO1-mediated tryptophan depletion that induces T cell proliferative arrest and promotes immunosuppressive Treg expansion; and hypoxia-amplified ROS accumulation that compounds mitochondrial dysfunction and accelerates TCR signaling erosion. The intersecting nature of these mechanisms explains why single-pathway interventions—including PD-1/PD-L1 blockade—achieve only partial and transient responses, and underscores the rationale for multi-target metabolic checkpoint strategies. These mechanisms are summarized in **Figure 1** and **Table 1**.

**Table 1:** HPV oncoprotein metabolic targets and their consequences for tumor metabolism and immune suppression.

HPV oncoprotein/effector	Metabolic target/pathway	Effect on tumor metabolism	Immune consequence and evidence grade (refs)
E6/E7 → GLUT1, HK2	Glucose uptake; first committed glycolytic step (Warburg effect)	Increased glucose consumption and lactate output	Starves TILs of glucose. Grade: HPV cervical tumor-cell-only (5, 10, 12).
E6/E7 → IGF2BP2/m^6^A-MYC	m^6^A-dependent MYC mRNA stabilization	Sustained high-flux glycolysis independent of HIF-1α	Heightens nutrient competition. Grade: HPV cervical tumor-cell-only (5).
E6 → VHL/HIF-1α	Attenuates VHL–HIF-1α interaction, stabilizing HIF-1α	Upregulates glycolytic genes; supports Warburg phenotype	Drives PD-L1 via HIF-1α. Grade: HPV cervical tumor-cell-only (27).
Glycolysis → MCT4 lactate export	Lactate efflux and extracellular acidification	Low pH; lactate as a signaling/epigenetic substrate	Impairs TIL glycolysis; supports Treg. Grade: mixed/context-dependent (17, 19, 20).
IFN-γ/inflammation → IDO1	Tryptophan → kynurenine catabolism	Tryptophan depletion; kynurenine accumulation	TIL arrest; AHR-driven PD-1/Treg. Grade: broadly supported (23–26).
Hypoxia/iron dysregulation	HIF-1α/PD-L1; TfR1 up, ferroportin down (proposed)	ROS accumulation; expanded labile iron pool	PD-L1 up; sensitizes TILs to ferroptosis. Grade: mechanistic extrapolation (27).

The final column gives an evidence grade (direct HPV cervical + immune model; HPV cervical tumor-cell-only; non-HPV cancer model; or mechanistic extrapolation) and supporting references.

### 2.6 Cervicovaginal and gut microbiota and their metabolites

Beyond the tumor-cell-intrinsic axes described above, accumulating evidence indicates that the local microbiota and their metabolites help shape the metabolic and immune landscape of HPV-associated cervical disease. A healthy cervicovaginal niche is typically dominated by Lactobacillus species; loss of this dominance and a shift toward a diverse, anaerobe-rich community (e.g., Gardnerella, Sneathia, Atopobium/Fannyhessea and related taxa) is associated with persistent HR-HPV infection, accelerated progression to cervical intraepithelial neoplasia and invasive cancer, a more immunosuppressive microenvironment, and—in emerging cohort studies—poorer treatment response and prognosis. Mechanistically, microbiota-derived metabolites may reinforce several of the metabolic checkpoints discussed in this review: organic acids and amine catabolites can modulate local pH and redox tone; bacterial and host co-metabolism of tryptophan generates indole derivatives and other aryl-hydrocarbon-receptor (AHR) ligands that intersect the IDO1–kynurenine–AHR axis; and short-chain fatty acids (notably butyrate) act as histone deacetylase inhibitors and immunometabolic signals that can influence regulatory T cell and CD8^+^ T cell programming. Dysbiosis-associated lipopolysaccharide and other pattern-associated molecules can additionally sustain chronic inflammation and IDO1 induction. Most of these links remain correlative or derive from non-cervical mucosal and gut models, and causal, mechanistic data directly connecting specific cervicovaginal taxa or metabolites to CD8^+^ TIL metabolism in HPV-positive cervical cancer remain limited; the microbiota nonetheless represents a biologically plausible, potentially modifiable upstream node that warrants integration into future immunometabolic studies and is discussed further as a translational consideration.

## 3 Intrinsic metabolic mechanisms of T cell exhaustion

The TME metabolic perturbations described above do not merely impose external constraints on TILs; they compel T cells to undergo maladaptive intrinsic metabolic rewiring in a failed attempt to survive under chronic antigen stimulation. This section delineates three interconnected intracellular mechanisms that collectively drive irreversible terminal exhaustion: glycolytic flux suppression through PD-1/mTORC1 uncoupling, mitochondrial fragmentation and impaired biogenesis, and lipid peroxidation-driven ferroptosis. These intrinsic dysfunctions are not independent events but form a progressive metabolic collapse—each layer amplifying the next—that ultimately renders TILs refractory to ICB.

### 3.1 Suppression of glycolytic flux

The synthesis of cytotoxic effector molecules—perforin, granzymes, IFN-γ—and the sustained killing capacity of effector T cells depend critically on mTORC1-driven aerobic glycolysis. Yet in HPV-associated cervical lesions and chronically infected tissue, infiltrating CD8^+^ TILs exhibit a characteristic glycolytic deficit attributable to blockade of upstream signaling and transcriptional downregulation of key metabolic enzymes. Bioenergetic insufficiency arising from PD-1-mediated metabolic alterations has been identified as an early driver of CD8^+^ T cell exhaustion, preceding the acquisition of a fully exhausted transcriptional phenotype (30).

Mechanistically, PD-1 engagement recruits the phosphatase SHP-2, which dephosphorylates critical serine and threonine residues within the PI3K/Akt signaling axis, blocking mTORC1 activation and its downstream effector programs (31). Sustained mTORC1 deficiency prevents phosphorylation of its direct substrates S6K1 and 4E-BP1, suppressing translational synthesis of HIF-1α protein. During this early, PD-1-driven phase, reduced HIF-1α abundance can downregulate GLUT1 and HK2 transcription, contributing to diminished glucose uptake and glycolytic throughput that deprives effector T cells of the aerobic glycolysis required for effector function (32). However, a simple “low HIF-1α equals exhaustion” model is an oversimplification: the relationship between HIF-1α and exhaustion appears to be stage-dependent. Whereas early PD-1 engagement suppresses mTORC1-dependent HIF-1α translation and effector glycolysis, later terminal exhaustion is characterized by a distinct, maladaptive remodeling: mitochondrial dysfunction can paradoxically stabilize HIF-1α and drive HIF-1α-mediated glycolytic reprogramming that promotes the precursor-to-terminal exhaustion transition (32). The metabolic trajectory of exhausting TILs is therefore better viewed as a dynamic sequence—early nutrient and signaling restriction, compensatory mitochondrial stress, maladaptive HIF-1α/glycolytic rewiring in terminal exhaustion, and eventual lipid peroxidation or ferroptosis in susceptible populations—rather than as a monotonic decline in glycolytic capacity.

Intrinsic transcriptional negative-feedback mechanisms further exacerbate this glycolytic deficit. The transcriptional repressor BACH2 binds specifically to effector gene enhancer regions and competitively antagonizes AP-1 family transcription factors; under conditions of chronic antigen stimulation, sustained BACH2 activity continuously blocks TCR-downstream glycolytic programming, preventing T cells from entering the high-glycolytic effector state and instead maintaining a mitochondria-dependent, low-effector metabolic phenotype that progressively drives terminal exhaustion (33).

### 3.2 Mitochondrial dynamics dysregulation and impaired biogenesis

Following uncoupling of the glycolysis–mTORC1 axis, T cells would theoretically upregulate oxidative phosphorylation (OXPHOS) to sustain ATP homeostasis. However, the HPV TME imposes a triple assault on mitochondrial integrity: chronic hypoxia suppresses mitochondrial fusion by promoting proteolytic degradation of fusion machinery; persistent antigen stimulation phosphorylates and activates the fission GTPase Drp1; and oxidative stress damages inner membrane structures. These converging forces produce a profound imbalance in mitochondrial dynamics that abolishes the bioenergetic compensation that might otherwise maintain T cell viability.

Yu et al. (34) demonstrated that exhausted CD8^+^ TILs exhibit a characteristic punctate, fragmented mitochondrial morphology. Mechanistically, chronic low-level TCR stimulation phosphorylates Drp1 at serine 616, promoting its recruitment to the outer mitochondrial membrane and driving excessive fission. Concurrently, persistent hypoxia in the tumor microenvironment promotes proteolytic degradation of the fusion proteins OPA1 and MFN1/2, with consequent downregulation of their expression (35). The resulting imbalance between fission and fusion leads to mitochondrial fragmentation, disruption of cristae architecture, and impairment of electron transport chain (ETC) supercomplex assembly—structural damage that reduces ATP synthesis rates, promotes electron leakage from the respiratory chain, and generates excessive ROS.

Impaired mitochondrial biogenesis constitutes a second, equally critical driver of T cell exhaustion, governed by the transcriptional coactivator PGC-1α. In the HPV-induced chronic inflammatory microenvironment, PGC-1α expression is subject to stringent negative regulation. Scharping et al. (36) demonstrated that combined persistent antigen stimulation and hypoxia induces overexpression of the transcriptional repressor Blimp-1, which directly binds the Ppargc1a promoter, recruits epigenetic modifying enzymes, and silences PGC-1α transcription. PGC-1α loss triggers dual pathological consequences: reduced expression of the mitochondrial transcription factor TFAM diminishes mitochondrial DNA replication and overall mitochondrial mass, while concurrent suppression of PGC-1α-dependent antioxidant enzyme programs further erodes cellular ROS defenses. Vardhana et al. (37) showed that this antioxidant insufficiency allows ROS generated by damaged mitochondria to accumulate unchecked, activating sustained DNA damage response pathways that withdraw T cells from the cell cycle and drive irreversible metabolic senescence.

### 3.3 Lipid peroxidation accumulation and ferroptosis

Recent advances in immunometabolism have identified lipid metabolic reprogramming as a critical and largely irreversible driver of T cell exhaustion in the HPV TME. HPV-associated tumor tissue is characterized by a lipid-rich microenvironment generated by accelerated tumor lipogenesis and extracellular lipid accumulation. Theoretically, these abundant extracellular lipids could serve as alternative carbon sources for TILs through mitochondrial fatty acid β-oxidation, compensating for the glycolytic deficit imposed by glucose competition. In practice, this metabolic rescue fails: the same mitochondrial fragmentation and bioenergetic collapse described above abolishes β-oxidation capacity in exhausted TILs, rendering imported fatty acids not a fuel source but a toxic liability. This bioenergetic trap—where alternative substrates cannot be utilized because the machinery to process them is structurally broken—sets the stage for the lipotoxic cascade that ultimately drives ferroptotic cell death.

Under chronic HPV antigen stimulation, CD8^+^ T cells compensatorily upregulate the scavenger receptor CD36 in a failed attempt to import extracellular lipids as alternative fuel. Xu et al. (38) demonstrated that CD36 overexpression drives excessive uptake of oxidized low-density lipoprotein and long-chain polyunsaturated fatty acids (PUFAs) from the tumor-conditioned lipid environment. Once internalized, oxidized lipids activate p38 MAPK stress signaling and trigger the unfolded protein response, compounding the mitochondrial damage already established above. Critically, because β-oxidation is impaired in structurally fragmented mitochondria, imported PUFAs cannot be channeled into ATP-generating oxidative metabolism; instead, acyl-CoA synthetase long-chain family member 4 (ACSL4) esterifies free PUFAs into membrane phospholipids, creating an expanded pool of peroxidation-susceptible phospholipid species that primes the cell for ferroptosis (39). This lipid-loading process simultaneously disrupts TCR immunological synapse assembly and pathologically raises the antigen-recognition threshold, directly impairing antiviral effector cytotoxicity even before cell death occurs. Notably, the relationship between mitochondrial dysfunction and lipid peroxidation is bidirectional and self-amplifying: ROS generated by damaged mitochondria initiate lipid radical chain reactions, while accumulating phospholipid hydroperoxides in turn further damage mitochondrial inner membranes—an escalating cycle that accelerates the transition from functional exhaustion to irreversible ferroptotic death.

The terminal event in this cascade is ferroptosis—an iron-dependent, regulated cell death modality driven by unrestricted phospholipid hydroperoxide accumulation. Two converging mechanisms disable the principal ferroptosis defense in exhausted TILs. First, HPV-transformed tumor cells competitively deplete extracellular cystine, the obligatory precursor for intracellular glutathione (GSH) synthesis; GSH depletion inactivates glutathione peroxidase 4 (GPX4), the enzyme responsible for reducing phospholipid hydroperoxides to non-toxic hydroxyl species. Second, the ACSL4-expanded membrane PUFA pool saturates residual GPX4 catalytic capacity with peroxidation substrates, overwhelming even partial antioxidant defenses. Ma et al. (40) directly demonstrated that CD36-mediated lipid peroxidation drives ferroptotic death in intratumoral CD8^+^ T cells *in vivo*, impairing their antitumor function, and that GPX4 restoration rescues TIL viability and effector capacity. The labile iron pool required to catalyze ferroptosis-initiating Fenton reactions is further augmented in the HPV TME: HPV-associated oncoproteins have been proposed to dysregulate host iron metabolism genes—including upregulation of transferrin receptor and suppression of ferroportin—amplifying intracellular free iron and cooperatively sensitizing TILs to ferroptotic stimuli, though direct mechanistic evidence in T cells specifically remains to be established (41).

**Figure 2:**
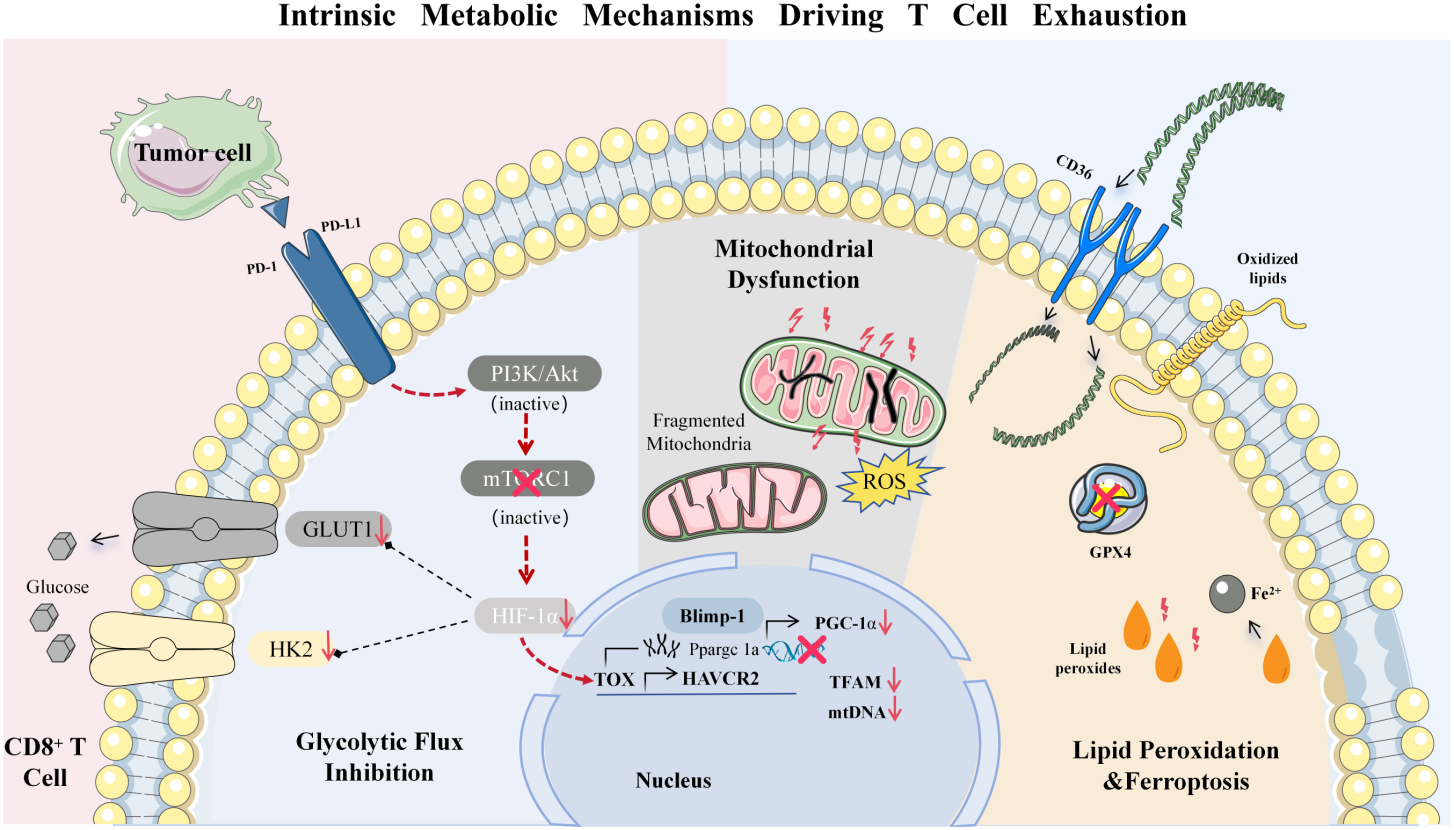
Molecular mechanisms of T-cell metabolic dysfunction and exhaustion in response to microenvironmental stress. PD-1 engagement recruits SHP-2 to dephosphorylate PI3K/Akt, blocking mTORC1 and suppressing HIF-1α-dependent GLUT1/HK2 transcription, thereby eliminating aerobic glycolysis. Chronic antigen stimulation drives Drp1-mediated mitochondrial fission while hypoxia promotes OPA1/MFN degradation, causing fragmentation, ETC disassembly, and ROS overproduction; Blimp-1-mediated PGC-1α silencing further suppresses mitochondrial biogenesis. In the lipid-rich TME, CD36-mediated PUFA uptake with GPX4 inactivation drives phospholipid peroxidation and ferroptosis. These axes converge to produce terminal T cell exhaustion. Evidence note: these pathways derive mainly from non-HPV tumor and chronic-infection models and represent a proposed, stage-dependent sequence; the low-HIF-1α→reduced-glycolysis step applies to the early PD-1-driven phase, whereas in terminal exhaustion mitochondrial dysfunction can instead stabilize HIF-1α.

Taken together, infiltrating T cells in the HPV TME undergo a progressive metabolic collapse spanning three interconnected levels: PD-1/mTORC1-mediated glycolytic suppression that eliminates the aerobic glycolysis underpinning effector function; mitochondrial fragmentation and PGC-1α silencing that abolish bioenergetic reserve and antioxidant capacity; and CD36-driven lipid peroxidation culminating in GPX4-dependent ferroptosis. The sequential and mutually reinforcing nature of these intrinsic dysfunctions explains why T cell exhaustion in the HPV TME is not merely a quantitative reduction in effector activity but a qualitatively distinct, epigenetically and metabolically consolidated state that resists reversal by ICB alone. The molecular mechanisms of T cell metabolic dysfunction are illustrated in **Figure 2**.

### 3.4 Metabolic distinctions among exhausted, senescent, and terminally differentiated T cells

“Dysfunctional” TILs are not a single homogeneous population; exhaustion, senescence, and terminal effector differentiation are distinct states with overlapping but separable metabolic signatures. Classical TEX arises under chronic antigen and metabolic stress and is defined by sustained inhibitory-receptor expression (PD-1, TIM-3, LAG-3), TOX-driven epigenetic programming, and the progressive bioenergetic collapse detailed above—impaired glycolysis, fragmented and depolarized mitochondria with low spare respiratory capacity, PGC-1α silencing, and susceptibility to lipid peroxidation and ferroptosis. Senescent T cells, by contrast, are typically driven by replicative or stress-induced signals rather than persistent TCR engagement; they characteristically lose CD27/CD28, re-express CD57 and KLRG1, show telomere attrition and stable cell-cycle arrest (p16/p21), and adopt a senescence-associated secretory phenotype. Metabolically, senescent T cells often retain or even increase glycolytic and mitochondrial mass while accumulating dysfunctional mitochondria and ROS, with AMPK/p38-MAPK and altered autophagy as prominent nodes—contrasting with the bioenergetic insufficiency that typifies exhaustion. Terminally differentiated effector and effector-memory cells (including TEMRA) represent a third state: short-lived, highly cytotoxic, glycolysis-skewed cells that need not display the inhibitory-receptor/TOX program of exhaustion. These distinctions matter clinically: interventions that rebuild mitochondrial fitness (e.g., PGC-1α or SIRT1 activators) are most rationally directed at exhausted cells, whereas senescence is more likely to require senolytic or secretory-phenotype-targeting strategies; the same metabolic drug may thus help one population and be neutral or harmful to another. Much of this framework derives from chronic-infection and non-cervical tumor models, and the relative contributions of exhaustion versus senescence among HPV-specific cervical TILs have not been systematically resolved and await single-cell metabolic profiling.

## 4 Natural product-based metabolic checkpoint intervention strategies

Glucose deprivation, lactate-driven acidification, IDO1-mediated tryptophan depletion, and lipid peroxidation collectively constitute the multi-layered metabolic barriers that render TILs dysfunctional in the HPV TME. Natural products offer a pharmacologically distinct approach to dismantling these barriers: rather than direct cytotoxicity, bioactive phytochemicals modulate specific metabolic enzymes or signaling nodes in a context-dependent manner—targeting the tumor metabolic compartment to relieve nutrient competition, or directly rehabilitating TIL mitochondrial fitness and antioxidant defenses. The following sections evaluate the mechanistic evidence for representative agents, organized by their primary metabolic target axis, and explicitly identify the compartment (tumor or immune) in which each compound exerts its primary documented effect.

### 4.1 Targeting Warburg effect-driven glucose competition

#### 4.1.1 Ginsenosides

Ginsenosides are a class of triterpenoid saponins extracted from Panax ginseng with broad antitumor and immunomodulatory activity. Two structurally distinct members target the glycolytic pathway through complementary mechanisms. Li et al. (42) demonstrated that ginsenoside Rg3 specifically blocks STAT3 phosphorylation at Tyr705, transcriptionally downregulating HK2 mRNA and protein expression, reducing tumor-cell glucose affinity and indirectly preserving extracellular glucose for TIL utilization. Shin et al. (43) showed that ginsenoside F2 activates miR-193a-5p expression downstream of c-Myc, suppressing the β-catenin/c-Myc/HK2 signaling axis and downregulating HK2, PKM2, and LDH-A protein levels in HPV-positive cervical cancer cells—directly reducing glucose uptake and lactate production. A key advantage of both mechanisms is their reliance on tumor-specific signaling nodes (STAT3, c-Myc), which are less active in mature T cells, theoretically preserving the T cell capacity to upregulate GLUT1 upon antigen-driven activation. Direct T cell metabolic validation in immune-competent co-culture or *in vivo* models is currently lacking.

#### 4.1.2 Shikonin

Shikonin (5,8-dihydroxy-2-[(1R)-1-hydroxy-4-methylpent-3-en-1-yl]naphthalene-1,4-dione), a naphthoquinone extracted from Lithospermum erythrorhizon, acts as an allosteric inhibitor of pyruvate kinase M2 (PKM2)—the glycolytic isoform preferentially expressed in tumor cells to accumulate upstream biosynthetic intermediates. Zhao et al. (44) demonstrated that shikonin inhibits PKM2-mediated aerobic glycolysis, blocking the terminal glycolytic step, depleting tumor ATP supply, and reducing lactate generation and export, thereby attenuating microenvironmental acidification. Notably, the structural picture is more complex than a simple dimeric conformational lock: shikonin has been reported to inhibit both dimeric and tetrameric PKM2 and to promote its aggregation, so the precise allosteric mechanism in this context should not be overstated.

However, this strategy carries a significant immunometabolic caveat. T cells require a switch to aerobic glycolysis during early antigen activation to support the rapid biosynthetic demands of clonal expansion and effector differentiation (45); that study demonstrated that aerobic glycolysis promotes Th1 differentiation through an epigenetic mechanism rather than a PKM2-specific dependence in T cells, so the inferred PKM2 requirement in activating T cells is an extrapolation (45). Non-selective PKM2 inhibition risks impairing the glycolytic reprogramming essential for T cell activation. This consideration argues for temporal restraint: shikonin-based glycolytic blockade is most likely to benefit TILs already in an exhausted rather than an acutely activating state, and may be better deployed sequentially after initial ICB-driven T cell reactivation. Precise pharmacokinetic scheduling in immune-competent models is required to resolve this therapeutic window.

#### 4.1.3 Curcumin

Curcumin (1,7-bis(4-hydroxy-3-methoxyphenyl)-1,6-heptadiene-3,5-dione), a polyphenol extracted from Curcuma longa rhizomes, counteracts the HIF-1α-driven Warburg phenotype that HPV oncoproteins actively reinforce in cervical cancer cells. Mechanistically, HPV16 E6 oncoprotein directly binds HIF-1α and physically disrupts its interaction with the VHL E3 ubiquitin ligase, attenuating VHL-mediated HIF-1α polyubiquitination and proteasomal degradation, thereby stabilizing HIF-1α and amplifying downstream glycolytic gene transcription (46). Curcumin counters this oncoviral subversion by restoring VHL–HIF-1α binding: it upregulates VHL protein expression, reactivates HIF-1α ubiquitin-proteasomal degradation, and consequently suppresses transcription of HIF-1α target glycolytic genes (47).

In cervical cancer cells specifically, curcumin suppresses the mTOR/HIF-1α axis to downregulate PKM2, reducing tumor-cell viability through Warburg effect inhibition (48). Downstream of HIF-1α degradation, curcumin indirectly downregulates GLUT1, HK2, LDHA, and PDK1, reducing glucose uptake and lactate release. Quantitative proteomics further revealed direct binding to multiple glycolytic enzymes and concurrent downregulation of MCT1 and MCT4, blocking lactate export (49). The consequent reduction in microenvironmental acidification decreases H3K18la and HIF-1α-dependent PD-L1 expression, together providing an indirect immune benefit. These multi-target immunomodulatory effects make curcumin one of the stronger candidates in the tumor-compartment-targeting category, though simultaneous measurement of T cell metabolic recovery remains to be demonstrated.

#### 4.1.4 Quercetin

Quercetin (3,3′,4′,5,7-pentahydroxyflavone) is a flavonoid abundant in fruits and vegetables. It targets the terminal lactate export step to ameliorate TME acidification and indirectly restore CD8^+^ T cell effector function. Quercetin suppresses glycolysis via Akt-mTOR pathway inhibition and autophagy induction, downregulating PKM2, GLUT1, and LDHA expression and reducing glucose uptake and lactate production (50); this Akt-mTOR/glycolysis mechanism was characterized primarily in breast cancer cells, so its applicability to cervical cancer is, at this point, inferred. Pani et al. (51) confirmed dose-dependent reductions in glucose uptake and lactate generation in HeLa cervical cancer cells. Quercetin additionally inhibits MCT1 and MCT4 expression, blocking tumor-cell lactate export (52), synergistically attenuating microenvironmental acidification and the downstream histone H3K18 lactylation-mediated epigenetic silencing of TIL effector genes.

This lactate-clearance strategy, however, harbors a therapeutically important paradox. Non-selective MCT1 blockade impairs T cells’ own ability to export glycolysis-derived lactate, causing intracellular acidification that inhibits phosphofructokinase and suppresses T cell glycolytic flux (53). Compounding this complexity, lactate is not a uniformly immunosuppressive molecule: Faubert et al. (54) showed that lactate serves as a carbon source for TCA cycle oxidative metabolism in tumors; Angelin et al. (55) demonstrated that Tregs exploit Foxp3-dependent metabolic reprogramming to preferentially utilize lactate; and Feng et al. (56) reported that physiological lactate concentrations maintain CD8^+^ T cell stemness through histone lactylation. Indiscriminate lactate elimination may therefore impair beneficial antitumor T cell subsets. These considerations argue that the therapeutic objective should be reducing lactate to within an immunostimulatory range through transcriptional modulation of MCT expression—as curcumin achieves via HIF-1α/mTOR downregulation—rather than acute channel blockade.

### 4.2 Restoring mitochondrial bioenergetics in exhausted TILs

Mitochondrial dysfunction—fragmentation, impaired biogenesis, and ROS accumulation—is the bioenergetic foundation of terminal T cell exhaustion. Agents in this category act primarily on the T cell immune compartment to restore oxidative phosphorylation capacity, rather than targeting tumor glycolysis. Their immune benefit is therefore more direct than that of the tumor-glycolysis–targeting agents, though few have been tested in HPV-specific *in vivo* immune models. Notably, physiological mitochondrial ROS serve as necessary second messengers for T cell activation (57); natural products in this category should therefore be framed as redox homeostasis modulators rather than generic antioxidants.

#### 4.2.1 Berberine

Berberine, an isoquinoline alkaloid extracted from Coptis chinensis and Phellodendron amurense, is the strongest dual-compartment candidate evaluated in this review, with preliminary evidence of acting on both the tumor metabolic compartment and the T cell immune compartment. HPV-positive cervical cancer cells show heightened berberine sensitivity relative to HPV-negative cells, attributable to the dependence of HPV E6/E7-transformed cells—whose pRB-mediated cell cycle control is disrupted—on AMPK-mediated metabolic stress responses (58, 59).

Berberine induces mitochondrial membrane potential depolarization, activating AMPK Thr172 phosphorylation and triggering two parallel metabolic programs: first, mTORC1 suppression reduces HIF-1α protein synthesis and downregulates GLUT1, HK2, and LDHA transcription, decreasing tumor-cell glucose uptake and lactate generation (60, 61); second, AMPK-mediated PGC-1α phosphorylation promotes TFAM and NRF1 expression, restoring fused tubular mitochondrial morphology and oxidative phosphorylation function in exhausted CD8^+^ T cells (62). This dual-compartment activity is assembled from cross-context evidence rather than from direct HPV-positive cervical cancer TIL studies: the AMPK–PGC-1α mitochondrial-biogenesis data derive largely from osteoblasts and other non-immune or non-cervical models, while the tumor glycolytic-suppression data come from colon and other cancer cell lines. Simultaneous validation of both effects within the same HPV-specific immune-competent system has not yet been performed, so berberine’s “dual-compartment” designation here is best read as a mechanistically plausible hypothesis. This dual activity is mechanistically coherent because the same AMPK activation that blocks tumor HIF-1α simultaneously activates the PGC-1α axis in T cells. An important caveat applies, however: exhausted TILs exhibit chronic GLUT1 downregulation (63), and in the absence of additional signals promoting GLUT1 membrane translocation, T cells may remain unable to recapture glucose even when microenvironmental availability is restored by berberine’s tumor glycolytic suppression. This gap reinforces the rationale for combining berberine with ICB, which can restore TCR-proximal signaling that drives GLUT1 surface expression.

#### 4.2.2 Resveratrol

Resveratrol (3,5,4′-trihydroxystilbene), a stilbenoid polyphenol, acts primarily on the T cell immune compartment through the SIRT1/PGC-1α axis. Unlike the glycolysis-targeting compounds, resveratrol does not directly suppress tumor glucose metabolism. Instead, it activates SIRT1-mediated PGC-1α deacetylation, promoting mitochondrial DNA replication and respiratory chain complex assembly, restoring oxidative phosphorylation capacity in CD8^+^ T cells (64). This mechanism is particularly relevant to HPV chronic infection, where persistent antigen stimulation drives mitochondrial fragmentation and PGC-1α transcriptional silencing. By rebuilding fused tubular mitochondrial networks, resveratrol elevates spare respiratory capacity and delays the transition to terminal exhaustion (65).

A critical therapeutic window constraint applies: effector T cells during acute activation depend on high-flux glycolysis to support rapid proliferation and effector molecule synthesis. Excessive SIRT1 activation at this stage could suppress mTORC1 signaling and impair granzyme B synthesis (66, 67). Resveratrol is therefore better positioned as a maintenance agent to promote exhausted-to-memory T cell conversion after initial ICB-driven reactivation, rather than to amplify acute cytotoxic function.

#### 4.2.3 Sodium selenite

Sodium selenite (Na_2_SeO_3_), an inorganic selenium compound, induces selective mitophagy in HPV-associated cervical cancer cells. In HeLa and SiHa cells, it disrupts tumor-cell energy balance, inhibits glucose uptake, activates AMPK phosphorylation, suppresses mTOR activity, and promotes FOXO3a nuclear translocation (68). FOXO3a, a key autophagy transcription factor, upregulates mitophagy-related gene expression, selectively clearing ROS-damaged mitochondria and inducing tumor-cell apoptosis. Sodium selenite’s primary documented action is tumor-cell directed; its selenium cytotoxicity limits clinical dosing. A rational combination strategy pairs low-dose sodium selenite (mitophagy induction in tumor cells) with berberine (mitochondrial biogenesis in T cells) to achieve complementary mitochondrial quality control in the respective compartments.

#### 4.2.4 Ficus carica extract

Ficus carica Linn. latex extract (FCE) selectively induces apoptosis in HPV-positive cervical cancer cells through the intrinsic mitochondrial pathway. In HPV18-positive HeLa and HPV16-positive SiHa cells, FCE triggers mitochondrial membrane potential collapse, cytochrome c release, and caspase cascade activation, with comparatively lower toxicity toward normal cells (69). Additionally, FCE reduces tumor-cell ROS generation and upregulates MHC class I expression, enhancing T cell immunological recognition (70). FCE’s primary benefit is tumor-compartment directed. Its active components—bergapten and psoralen—have known photosensitizing properties that may offer synergy with photodynamic therapy approaches targeting tumor mitochondria, though this remains speculative and requires experimental validation. FCE currently lacks direct evidence of T cell metabolic rehabilitation and should be considered a tumor-targeting agent.

### 4.3 Targeting tryptophan catabolism and lipid peroxidation

The IDO1–kynurenine–AHR axis and the CD36-driven lipid peroxidation–ferroptosis axis represent distinct biochemical immunosuppressive barriers that operate independently of glucose competition and lactate acidification. Natural products targeting these pathways act more directly on the T cell immune compartment—either restoring tryptophan availability to relieve T cell proliferative arrest, or protecting TIL membrane integrity from lipid peroxide-driven ferroptosis.

#### 4.3.1 EGCG (targeting IDO1-mediated tryptophan depletion)

(−)-Epigallocatechin-3-gallate (EGCG), the principal bioactive catechin in green tea, has been identified as a potent natural IDO1 inhibitor. Cheng et al. (71) demonstrated that EGCG competitively binds the IDO1 active site to directly inhibit its enzymatic activity, and additionally blocks the JAK/PKC-δ/STAT1 signaling cascade to downregulate IDO1 at the transcriptional level. By preserving tryptophan availability, EGCG deactivates GCN2-mediated integrated stress response in T cells, relieving proliferative arrest and restoring mTORC1-dependent anabolic programs. Concurrently, reduced kynurenine accumulation limits AHR activation, attenuating both PD-1 upregulation on CD8^+^ T cells and kynurenine-driven Treg differentiation from CD4^+^ precursors. EGCG thus acts on the TME amino acid compartment with demonstrable downstream benefit to T cell function. A key limitation is that single-target IDO1 blockade can trigger compensatory upregulation of TDO2 (tryptophan 2,3-dioxygenase) or downstream AHR ligands; combining EGCG with PD-1/PD-L1 antibodies may achieve synergistic immune reactivation by simultaneously relieving metabolic and receptor-mediated checkpoints.

#### 4.3.2 Baicalein (protecting TILs from ferroptosis)

Baicalein (5,6,7-trihydroxyflavone), a flavonoid from Scutellaria baicalensis, is a characterized inhibitor of 12/15-lipoxygenase (LOX) that acts directly on the T cell immune compartment to suppress ferroptosis. Xie et al. (72) identified baicalein as a potent ferroptosis inhibitor through natural product library screening, demonstrating that it scavenges phospholipid hydroperoxides and blocks the lipid peroxidation cascade. This study, however, characterized baicalein as a general ferroptosis inhibitor in a non-immune screening system rather than in CD8^+^ TILs specifically (72); its application to protecting CD8^+^ TIL GPX4 activity and membrane integrity in the HPV-associated TME is therefore an extrapolation that has not been directly validated in T cells. On this basis, baicalein is best described as a plausible ferroptosis-protective candidate whose proposed maintenance of GPX4 activity in CD8^+^ T cells requires direct experimental confirmation. The therapeutic rationale rests on the observation that HPV-positive tumor cells harbor abnormally expanded labile iron pools and overexpress transferrin receptor 1 (TfR1), rendering them substantially more sensitive to ferroptotic stimuli than T cells operating in the same TME. Targeted baicalein delivery to the immune compartment, or dual-agent strategies pairing a tumor-selective ferroptosis inducer with baicalein-mediated TIL protection, represents a tractable approach to exploiting this iron-load differential.

#### 4.3.3 Artemisinin derivatives (inducing tumor-cell ferroptosis)

Artemisinin-derived compounds, including artematrolide A (AR-A) isolated from Artemisia argyi and dihydroartemisinin (DHA), exert antitumor effects in cervical cancer through complementary metabolic mechanisms. AR-A suppresses HeLa and SiHa cell proliferation, migration, and invasion (IC_50_ at 24/48 h: 5.7/5.1 μM and 10.1/5.4 μM, respectively) by phosphorylating MEK/ERK, suppressing mTOR/HIF-1α expression, and activating pyruvate dehydrogenase complex (PDC) and α-ketoglutarate dehydrogenase complex (OGDC) activity to redirect pyruvate toward oxidative decarboxylation, reducing lactate generation and driving a metabolic shift from aerobic glycolysis to mitochondrial respiration that induces G_2_/M arrest and apoptosis (73).

DHA operates through a distinct ferroptosis-induction pathway. In HPV-positive HeLa and SiHa cells, DHA activates NCOA4-mediated ferritinophagy, promoting ferritin degradation and expanding the labile iron pool to trigger ROS burst and lipid peroxidation accumulation. DHA treatment markedly depletes GPX4 and glutathione (GSH), upregulates lipid peroxidation markers MDA and ACSL4, and its cytotoxicity is reversed by the ferroptosis inhibitor ferrostatin-1 but not by the apoptosis inhibitor Z-VAD-FMK, confirming ferroptosis as the dominant cell death modality (74). HPV-positive cervical cancer cells’ elevated TfR1 expression and expanded labile iron pool render them particularly susceptible to DHA-induced ferroptosis, and DHA demonstrates synergistic cytotoxicity with doxorubicin (75). Critically, the tumor-selective ferroptosis sensitivity created by HPV-driven iron dysregulation provides a potential therapeutic window: DHA preferentially induces tumor-cell ferroptosis at concentrations that may be tolerated by T cells with lower basal iron load. Combining DHA with baicalein-mediated TIL ferroptosis protection represents a mechanistically rational dual strategy that merits direct experimental evaluation.

**Table 2**, **Figure 3** summarize the molecular targets, compartment specificity, and evidence quality for each natural product discussed in this section. A unifying observation is that no single agent has been directly demonstrated to simultaneously rehabilitate both the tumor metabolic compartment and TIL function in HPV-specific immune-competent models; the complementary pharmacology described above remains largely inferred from independent tumor-cell and immune-cell studies. This evidence gap defines the primary experimental priority for translational development of natural product-based metabolic checkpoint strategies.

**Table 2:** Summary of natural product metabolic intervention targets, compartment specificity, and evidence quality.

Compound	Primary target	Metabolic effect	Compartment	Key evidence and evidence grade
Ginsenoside Rg3	STAT3 (Tyr705) → HK2	Inhibits tumor glycolysis; indirectly spares TME glucose for TILs	Tumor (indirect immune)	Ovarian cancer cells; no direct T cell metabolic data Grade: non-HPV cancer model (42).
Ginsenoside F2	miR-193a-5p → β-catenin/c-Myc/HK2	Downregulates HK2, PKM2, LDH-A in HPV+ cervical cells; reduces lactate	Tumor (indirect immune)	HPV+ cervical cancer cells; no immune co-culture data Grade: HPV cervical tumor-cell-only (43).
Shikonin	PKM2 (allosteric inhibition)	Blocks terminal glycolysis; reduces lactate; risks impairing T cell activation if non-selective	Tumor; T cell (risk)	Tumor-bearing mice; T cell PKM2 dependence in early activation Grade: non-HPV cancer model (tumor) + mechanistic extrapolation (T cell) (44, 45).
Curcumin	VHL/HIF-1α, mTOR/PKM2, MCT1/MCT4	Multi-target glycolytic suppression; reduces lactate, PD-L1, H3K18la; modulates Treg/Th1	Tumor (strongest multi-target); indirect immune	Cervical & colorectal cancer cells; proteomics; no direct T cell metabolic recovery data Grade: HPV cervical + non-HPV tumor-cell-only (46–49).
Quercetin	Akt-mTOR → PKM2/GLUT1/LDHA; MCT1/MCT4	Reduces glucose uptake & lactate in HeLa cells; MCT blockade paradox limits selectivity	Tumor (indirect immune); T cell (risk via MCT)	HeLa cervical; breast cancer cells; MCT paradox literature Grade: HPV cervical (HeLa) tumor-cell-only + non-HPV; T cell effect by extrapolation (50–56).
Berberine	AMPK → mTORC1/HIF-1α (tumor); AMPK → PGC-1α (T cell)	Suppresses tumor Warburg effect; promotes mitochondrial biogenesis in exhausted T cells via AMPK	Tumor + T cell (strongest dual-compartment candidate)	HPV+ cervical cells; colon cancer cells; AMPK/PGC-1α mitochondrial data Grade: HPV cervical tumor-cell-only (tumor) + mechanistic extrapolation, cross-context (T cell) (58–63).
Resveratrol	SIRT1/PGC-1α axis	Restores mitochondrial biogenesis and OXPHOS in exhausted T cells; no direct tumor glycolytic effect	T cell (direct)	Metabolic disease models (Lagouge 2006); mitochondrial fate T cell models; timing constraint Grade: non-HPV/mechanistic extrapolation; no HPV-specific TIL data (64–67).
EGCG	IDO1 (competitive inhibition + JAK/STAT1 transcriptional)	Restores TME tryptophan; deactivates T cell GCN2/ISR; reduces kynurenine-AHR-Treg axis	TME amino acid axis → T cell (indirect)	Human oral cancer cells; IDO1 enzyme kinetics Grade: non-HPV cancer model/mechanistic extrapolation (71).
Baicalein	12/15-LOX; phospholipid hydroperoxide scavenging; GPX4 maintenance	Protects CD8^+^ TILs from ferroptosis; maintains GPX4 activity; no tumor glycolytic effect	T cell (direct ferroptosis protection)	Natural product library screening; LOX inhibition assays Grade: mechanistic extrapolation (non-immune screen; not CD8^+^ TIL-specific) (72).
DHA/AR-A (Artemisinins)	NCOA4-ferritinophagy; GPX4/GSH depletion (DHA); mTOR/HIF-1α/PDC (AR-A)	Induces tumor-cell ferroptosis via labile iron expansion; reduces tumor glycolysis (AR-A)	Tumor (direct ferroptosis induction)	HPV+ HeLa/SiHa cells; ferrostatin-1 rescue; TfR1 overexpression in HPV+ cells Grade: HPV cervical tumor-cell-only (73–75).

HK2, hexokinase 2; PKM2, pyruvate kinase M2; LDH-A, lactate dehydrogenase A; HIF-1α, hypoxia-inducible factor 1-alpha; GLUT1, glucose transporter 1; MCT, monocarboxylate transporter; AMPK, AMP-activated protein kinase; PGC-1α, peroxisome proliferator-activated receptor gamma coactivator 1-alpha; OXPHOS, oxidative phosphorylation; IDO1, indoleamine 2,3-dioxygenase 1; AHR, aryl hydrocarbon receptor; ISR, integrated stress response; LOX, lipoxygenase; GPX4, glutathione peroxidase 4; DHA, dihydroartemisinin; AR-A, artematrolide A; TfR1, transferrin receptor 1; TME, tumor microenvironment; TIL, tumor-infiltrating lymphocyte. Note: The final column now provides a simple evidence grade for each compound and the supporting reference(s). Four grades are used: “direct HPV cervical + immune model” (the highest level of support, requiring data in HPV-positive cervical cancer together with an immune read-out); “HPV cervical tumor-cell-only” (evidence in HPV-positive cervical cancer cells but without immune validation); “non-HPV cancer model” (evidence in other tumor types); and “mechanistic extrapolation” (inference from general immunometabolism or non-immune assays). Notably, no compound in this table currently reaches the highest grade, underscoring that the proposed immune benefits remain largely inferred and require direct validation in HPV-specific, immune-competent systems.

**Figure 3:**
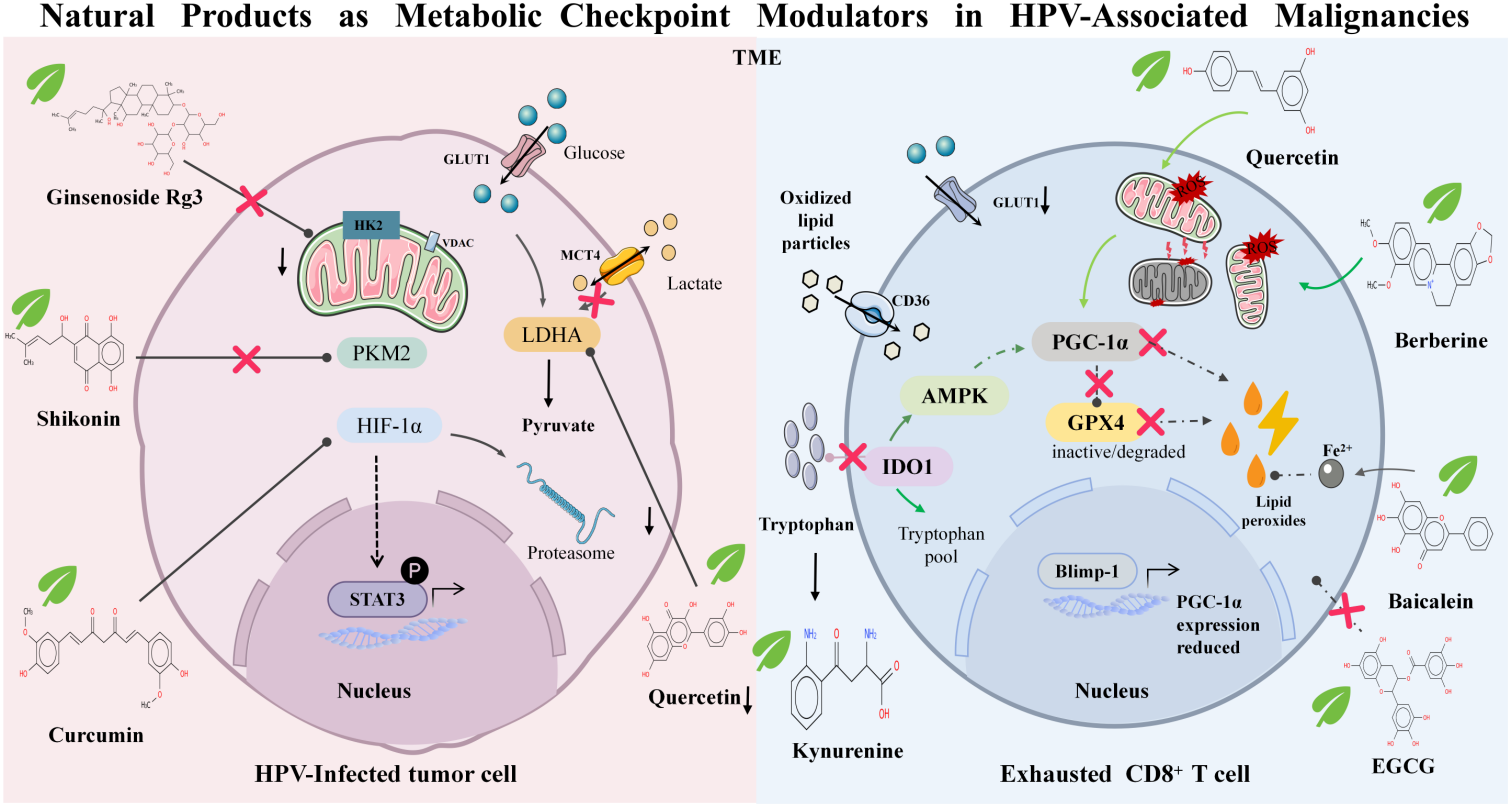
Compartment-specific metabolic intervention targets of natural products in the HPV-associated tumor microenvironment. The diagram stratifies natural-product targets into three axes. Tumor axis (left): ginsenosides (Rg3/F2), shikonin, curcumin, and quercetin suppress glycolytic enzymes (HK2, PKM2, LDHA) and lactate export (MCT4), relieving glucose competition and acidification. T cell mitochondrial axis (center): berberine and resveratrol activate AMPK–PGC-1α and SIRT1–PGC-1α, restoring mitochondrial biogenesis and OXPHOS in exhausted TILs. Tryptophan/lipid axis (right): EGCG inhibits IDO1 to restore tryptophan; baicalein protects TILs from ferroptosis; DHA induces tumor-selective ferroptosis. No single agent has shown simultaneous dual-compartment validation in HPV-specific immune-competent models. Abbreviations as in **Table 2**. Evidence levels for each intervention are graded in **Table 2**.

## 5 Challenges and future perspectives

Although natural products demonstrate compelling mechanistic potential for reshaping the immunosuppressive metabolic microenvironment in HPV-associated malignancies and reversing T cell exhaustion, the preponderance of supporting evidence derives from *in vitro* cell culture experiments or preclinical animal models. Three principal barriers must be overcome to translate these findings toward clinical application: pharmacokinetic limitations intrinsic to the physicochemical properties of most bioactive phytochemicals; the challenge of achieving therapeutic specificity within shared metabolic pathways; and the absence of validated metabolic biomarker frameworks to guide precision patient selection. Each of these challenges is examined below, with specific proposals for how they may be addressed.

### 5.1 Pharmacokinetic bottlenecks and intelligent delivery systems

Most natural polyphenols and alkaloids with immunometabolic regulatory activity are constrained by their inherent physicochemical properties, exhibiting poor aqueous solubility, limited chemical stability under physiological conditions, and rapid systemic clearance (76). Low oral bioavailability—stemming from extensive first-pass metabolism, efflux pump-mediated intestinal extrusion, and plasma protein binding—represents the primary factor limiting translation of *in vitro* potency to *in vivo* therapeutic efficacy. Developing smart delivery systems capable of traversing physiological barriers and exploiting tumor pathological features for site-specific drug accumulation is therefore critical to improving the therapeutic index of these compounds.

HPV-positive tumors generate substantial lactate via the Warburg effect, producing a local microenvironmental pH significantly below normal tissue (pH 6.5–6.8 vs. 7.4). This physicochemical signature can be exploited for targeted release. Hydrophobic glycolytic inhibitors such as shikonin and curcumin are candidates for encapsulation within polyhistidine-linked polymer nanocarriers that undergo protonation-triggered structural disassembly upon entering the acidic TME following enhanced permeability and retention (EPR)-mediated accumulation (77). This strategy concentrates drug payload at the tumor site while substantially reducing non-specific systemic toxicity toward normal organs.

For compounds targeting mitochondrial dysfunction and oxidative stress—such as baicalein and resveratrol—ROS-responsive nanogel delivery represents a complementary approach. Carriers incorporating disulfide crosslinks or thioether motifs undergo selective bond cleavage in the high-ROS environment of the TME, releasing antioxidant cargo precisely where lipid peroxidation-mediated TIL ferroptosis is occurring (78). This stimulus-responsive release profile simultaneously scavenges excess ROS and delivers ferroptosis-protective compounds to infiltrating T cells, achieving a spatiotemporal precision unattainable by systemic free-drug administration.

To circumvent reticuloendothelial system clearance and improve transmembrane delivery efficiency, biomimetic delivery platforms have emerged as a frontier strategy. Membrane-coated nanoparticles leverage natural cell surface components to achieve immune evasion and tumor-homing capabilities (79). Erythrocyte membrane-coated nanoparticles exploit camouflage-mediated clearance avoidance to substantially extend natural product plasma half-life, while platelet membrane-coated nanoparticles exploit the intrinsic inflammatory tropism of platelets to achieve active tumor homing and enhance lesional drug concentration (80). Beyond passive camouflage, herbal-component nanomedicines have been specifically engineered to prime antitumor immunity: by co-delivering bioactive phytochemicals with immunomodulatory cargo, such platforms can simultaneously remodel the metabolic microenvironment and reactivate effector lymphocytes, a design principle directly transferable to the metabolic checkpoint modulators discussed here (81). Experimentally supported nanomedicine examples must be distinguished from the speculative designs proposed here, which remain untested. One illustrative proposed—not yet realized—design pairs surface-functionalized anti-PD-L1 moieties (for physical checkpoint blockade) with an encapsulated PKM2 inhibitor such as shikonin. This concept carries an internal tension: as discussed above, non-selective PKM2 inhibition may impair the glycolytic reprogramming that T cells require during activation, so co-delivering a PKM2 inhibitor with an agent intended to re-activate T cells could be self-antagonistic unless release is spatially restricted to tumor cells and temporally separated from T cell re-activation. For this and every other proposed delivery system in this section, the intended cellular target, release trigger, expected pharmacokinetic advantage, anticipated immune toxicity, and the experimental model needed for validation should be specified before the design is pursued; in the present case the intended target is the tumor cell (not the T cell), the trigger would be the acidic/high-ROS TME, the expected advantage is tumor-restricted glycolytic suppression with reduced systemic exposure, the principal toxicity risk is on-target suppression of activating TILs, and validation would require an HPV-specific immune-competent model with pharmacokinetic and TIL-functional read-outs. These proposals should therefore be read as hypotheses requiring immune-competent, HPV-specific validation rather than as established strategies.

### 5.2 Therapeutic specificity and the metabolic intervention window

Aerobic glycolysis is not exclusively a tumor-cell metabolic program; it is equally indispensable for effector T cell cytokine synthesis, clonal expansion, and granzyme production during the activation phase. Non-selective inhibition of glycolysis by natural products thus carries an inherent risk of collateral suppression of activating T cells, potentially undermining the antitumor immune response that these compounds are intended to support. Resolving this therapeutic paradox requires exploiting micro-level differences in metabolic enzyme isoforms and adaptive flexibility between tumor cells and immune cells. HPV-transformed cells preferentially express the embryonic/tumor-specific isoforms PKM2 and HK2, whereas mature effector T cells rely substantially on distinct isoform profiles. Accordingly, natural product derivatives or formulations that selectively engage the allosteric regulatory sites unique to PKM2—rather than pan-kinase or pan-glycolytic activity—should theoretically suppress the tumor Warburg phenotype while preserving baseline T cell metabolic function (82).

T cell activation, differentiation, and exhaustion represent a dynamic metabolic continuum in which the cellular energy demands at each stage are fundamentally different, and the consequences of metabolic intervention vary accordingly. This has direct implications for clinical sequencing. During the induction phase, ICB agents should be administered to re-engage T cell receptor signaling and initiate clonal expansion; co-administration of glycolytic inhibitors at this juncture risks impairing the very metabolic reprogramming required for productive T cell activation (83). During the maintenance phase—after initial T cell expansion and transition to the effector state—introduction of natural product-based metabolic modulators targeting tumor glycolysis, IDO1 activity, or lipid peroxidation can deprive the tumor of its competitive metabolic advantage while simultaneously protecting against T cell re-exhaustion driven by the ongoing hostile microenvironment. This sequential framework requires prospective validation in immune-competent HPV-specific preclinical models with pharmacokinetic sampling to define the optimal transition timing between phases.

### 5.3 Biomarker-guided patient stratification for clinical trial design

Not all patients with HPV-associated malignancies will derive equivalent benefit from metabolic checkpoint intervention. Tumor metabolic phenotypes exhibit substantial inter-patient heterogeneity: a subset of cervical carcinomas relies predominantly on oxidative phosphorylation rather than glycolysis, rendering glycolytic inhibitors mechanistically irrelevant and potentially harmful in such cases. Prospective identification of patients most likely to benefit from metabolic intervention therefore requires validated companion biomarker strategies integrated into trial design from the outset.

A practical biomarker framework should operate on two complementary levels. At the tumor-tissue level, immunohistochemical quantification of HK2 and PKM2 protein abundance in pretreatment biopsy specimens—along with IDO1 expression for tryptophan pathway-targeting agents—can stratify patients by Warburg metabolic score and select those whose tumors are biochemically primed for the intended intervention. At the systemic level, plasma metabolomic profiling provides a minimally invasive, longitudinally trackable readout: elevated lactate-to-pyruvate ratio and kynurenine-to-tryptophan ratio (KTR) serve as functional surrogates for TME glycolytic intensity and IDO1 activity, respectively. Enrollment criteria in Phase I/II trials should prospectively define metabolic score thresholds, restricting natural product adjunct therapy to patients meeting pre-specified biomarker cutoffs. Equally important, pharmacodynamic endpoints in such trials should include on-treatment assessment of intratumoral CD8^+^ TIL metabolic functional markers—spare respiratory capacity, mitochondrial membrane potential, GPX4 expression, and H3K18 lactylation levels—to directly measure the immunometabolic remodeling effect and distinguish biological activity from clinical response.

The clinical positioning of natural product-based metabolic modulators should be explicitly framed as adjunctive to, rather than competitive with, standard ICB. The objective response rate of PD-1/PD-L1 inhibitors in advanced cervical cancer remains in the range of 15–25%, with resistance largely attributable to the multi-layered metabolic barriers documented above. Natural products that dismantle these metabolic barriers are rationally positioned to expand the fraction of patients deriving durable benefit from ICB, delay the onset of acquired resistance, and potentially enable dose reduction of immunotherapy agents in responders. Prioritized combination designs for near-term clinical investigation include: berberine plus pembrolizumab (targeting AMPK/mTORC1 dual-compartment activity alongside PD-1 blockade); EGCG plus PD-1 inhibitor (IDO1 suppression to relieve tryptophan depletion-mediated T cell arrest); and baicalein plus DHA (TIL ferroptosis protection paired with tumor-selective ferroptosis induction). Rigorous biomarker-stratified randomized Phase II trials are required to determine whether these combinations translate into improvements in progression-free survival and overall survival.

### 5.4 Translationally relevant metabolic checkpoints and the clinical trial landscape

The metabolic checkpoints relevant here are at different stages of clinical development, and the trials run so far show both their promise and their limits. The most clinically advanced immunometabolic target relevant to this review is the tryptophan–IDO1–kynurenine axis: small-molecule IDO1 inhibitors (e.g., epacadostat, navoximod, linrodostat) have been tested in combination with PD-1/PD-L1 blockade across multiple tumor types, and dual IDO1/TDO inhibitors have entered early-phase study. Importantly, the pivotal Phase III evaluation of epacadostat plus pembrolizumab (ECHO-301/KEYNOTE-252; ClinicalTrials.gov identifier NCT02752074) did not improve progression-free or overall survival over pembrolizumab alone in melanoma (84), a result that tempered enthusiasm for single-target IDO1 inhibition and underscores the need for rational biomarker selection (e.g., the kynurenine-to-tryptophan ratio) and combination logic rather than unselected use. Other metabolically defined checkpoints in active clinical development include the adenosinergic axis (CD73 and A2A receptor antagonists), lactate transport (the first-in-class MCT1 inhibitor AZD3965, evaluated in a Phase I trial in advanced solid tumors and lymphoma, NCT01791595) (85), and glutamine metabolism (glutaminase inhibitors such as telaglenastat), each of which intersects mechanisms discussed here. For HPV-associated cervical cancer specifically, dedicated trials that target these metabolic nodes and read out intratumoral T cell metabolic function remain scarce, and early-phase, biomarker-stratified studies in this indication are a priority.

For the natural products discussed here, the human evidence base is at an early stage and, critically, almost none of the existing trials were designed to read out immune-cell metabolic reprogramming. Several of these compounds have nonetheless entered clinical study in relevant settings: green-tea catechins/EGCG, formulated as Polyphenon E, were evaluated in a Phase II randomized, placebo-controlled trial in women with persistent high-risk HPV infection and low-grade cervical intraepithelial neoplasia (NCT00303823), which did not show significant promotion of HPV clearance or CIN1 regression over placebo (86); berberine reduced colorectal adenoma recurrence in a multicenter, double-blind, randomized controlled trial (NCT02226185) (87); and curcumin (including more bioavailable formulations), resveratrol, and quercetin have been studied largely for chemopreventive and metabolic endpoints. These trials have generally used clinical or histologic endpoints (lesion regression, HPV clearance, recurrence) rather than pharmacodynamic measures of TIL metabolism, and trials directly testing natural-product modulation of CD8^+^ T cell metabolic reprogramming in HPV-positive cervical cancer are, to our knowledge, lacking. These proposals therefore motivate future biomarker-embedded trials in which on-treatment changes in intratumoral TIL spare respiratory capacity, mitochondrial membrane potential, GPX4 expression, and H3K18 lactylation are incorporated as pharmacodynamic endpoints. Representative trials with verified ClinicalTrials.gov identifiers are summarized in **Table 3**.

**Table 3:** Representative clinical trials of natural products relevant to this review and their immunometabolic context.

Natural product (formulation)	Setting/indication (trial)	Design and primary endpoint	Outcome and immunometabolic relevance
Green-tea catechins/EGCG (Polyphenon E)	Persistent HR-HPV with low-grade CIN (NCT00303823) (86)	Phase II RCT; HPV clearance/CIN1 regression	No significant benefit vs placebo; no TIL metabolic endpoint
Berberine	Colorectal adenoma prevention (NCT02226185) (87)	Multicenter RCT; adenoma recurrence	Reduced recurrence; AMPK-linked metabolic rationale; no immune endpoint
Curcumin, resveratrol, quercetin	Chemoprevention/metabolic indications	Multiple early-phase studies; clinical or metabolic endpoints	Studied for chemopreventive/metabolic endpoints; no intratumoral T cell metabolic read-out

## 6 Conclusion

HPV E6/E7 oncoproteins are proposed to shape a metabolically hostile TME through four reinforcing axes—Warburg-driven glucose competition, lactate-mediated H3K18 lactylation, IDO1-dependent tryptophan catabolism, and hypoxia-amplified oxidative stress—that are thought to promote progressive, and in susceptible populations terminal, TIL exhaustion through PD-1/mTORC1 uncoupling, mitochondrial fragmentation, and GPX4-dependent ferroptosis. Because histone lactylation appears to contribute to chromatin-level consolidation of the exhaustion program in a manner that is at least partly independent of PD-1 signaling—while also exerting context-dependent, and in some settings stemness-supporting, effects—ICB alone is, in most patients, unlikely to restore productive antitumor immunity. The strength of evidence for individual steps in this model varies (see the evidence grades in **Tables 1** and **2**), and direct validation in HPV-positive cervical cancer TILs remains an important priority.

Bioactive phytochemicals offer compartment-specific metabolic intervention as adjuncts to ICB: berberine is the strongest dual-compartment candidate; curcumin and EGCG provide multi-target tumor glycolytic and tryptophan-axis suppression; and the baicalein/DHA combination exploits tumor-selective iron overload for differential ferroptosis modulation. Realizing the clinical potential of this strategy requires three priorities: isoform-selective formulations that spare T cell glycolysis during the activation phase; sequential ICB-then-metabolic modulator scheduling; and biomarker-stratified Phase II trials incorporating tumor HK2/IDO1 profiling and plasma metabolomic readouts as pharmacodynamic endpoints.
